# Recycling of Spent LiFePO_4_ Batteries Using Ultrasonic-Assisted Reducing Leaching

**DOI:** 10.3390/ma19143004

**Published:** 2026-07-13

**Authors:** Yi-Fan Gao, Rong-Liang Zhang, Jia-Xiang Liu, Ruo-Lan Ma, Wen Pan, Guang-Hui Fan, Li Tao

**Affiliations:** School of Metallurgy Engineering, Jiangsu University of Science and Technology, Zhangjiagang 215600, China

**Keywords:** spent lithium iron phosphate battery, cathode material, reducing leaching, ultrasonic-assisted leaching, battery recycling

## Abstract

The application of a huge number of lithium-ion batteries (LIBs) to electric vehicles has produced much solid waste. If not disposed properly, the solid waste may cause environmental pollution and is, per se, a waste of resources. Therefore, recycling valuable metals from LIBs is considered an ideal option for preventing environmental pollution and alleviating waste. Taking sulfuric acid (H_2_SO_4_) as the leaching agent and glucose (C_6_H_12_O_6_) as the reducing agent, the ultrasonic-assisted reducing leaching was used to recycle lithium (Li) and iron (Fe) from spent lithium iron phosphate (LFP) batteries. Based on experimental results of conventional leaching, the research aimed to examine the influence of ultrasonic treatment on leaching rates of Li and Fe. Results show that the leaching rates of Li and Fe are separately 96.53% and 96.8% when the concentration of H_2_SO_4_ is 2 mol/L, the concentration of C_6_H_12_O_6_ is 2 mol/L, the liquid–solid ratio is 15 mL/g, leaching temperature is 70 °C, leaching time is 60 min, and ultrasonic power is 100 W. Compared with conventional leaching, the leaching rates of Li and Fe separately increase by 10.84% and 12.33% through ultrasonic-assisted leaching under the same experimental conditions. Kinetics analysis of ultrasonic-assisted reducing leaching indicates that the activation energies of Li and Fe are 10.84 kJ/mol and 16.24 kJ/mol, respectively. The ultrasonic-assisted reducing leaching process of Li and Fe from LFP batteries is controlled by diffusion.

## 1. Introduction

Thanks to their high energy density, long service life, and comparatively light weight, lithium-ion batteries (LIBs) have become the power source of choice for portable electronics and, increasingly, electric vehicles. This rapid uptake has pushed battery production to unprecedented levels, and an equally rapid rise in the number of retired batteries entering the waste stream is now inevitable. Notably, spent LIBs contain concentrations of valuable metals well above those found in primary ores [1,2], and because these metals are already concentrated and chemically accessible, recovering them from battery waste is markedly less resource-intensive than mining and refining virgin ore [3]. Considering how much ore has already been consumed to manufacture today’s LIB fleet, recovering metals from end-of-life batteries offers a practical way to ease pressure on primary metal resources while advancing broader sustainability goals. There is also a safety dimension to this argument: batteries left untreated can rupture or corrode, releasing hazardous substances that pose a real risk to human health. Recycling spent LIBs therefore serves two purposes at once—capturing economic value and preventing environmental harm.

Among the technologies available for recovering these metals, hydrometallurgical processing has become the dominant approach for spent LiFePO_4_ (LFP) batteries, owing to its high selectivity, moderate energy requirements, and relatively low secondary pollution. A typical hydrometallurgical route begins by separating the active electrode material from its metal current collector—achieved by dissolving the polymer binder in an organic solvent, dissolving the aluminum foil in alkaline solution, or mechanically pulverizing the electrode assembly. The resulting cathode powder is then leached into a solution, most often with an inorganic acid such as HCl, H_3_PO_4_, or H_2_SO_4_ [4,5,6]; though organic acids including CH_3_COOH, H_2_C_2_O_4_, C_6_H_8_O_7_, and HCOOH have also proven effective [7,8,9,10], adding an oxidant such as H_2_O_2_, Na_2_S_2_O_8_, (NH_4_)_2_S_2_O_8_, or O_2_ can further boost extraction efficiency [11,12,13,14]. From the resulting leachate, iron and lithium are finally recovered as saleable salts via precipitation, solvent extraction, or concentration [15,16,17].

Reducing agents play a central role in this process, and glucose (C_6_H_12_O_6_) stands out among them for being inexpensive, non-toxic, and effective under mild reaction conditions—qualities that make it an appealing choice for hydrometallurgical applications. Its performance has been demonstrated in several cathode systems. Gao et al. combined 1.5 mol/L phosphoric acid with 0.02 mol/L glucose to leach cobalt and lithium from spent LIB cathode material, achieving recovery rates of 98% and nearly 100%, respectively [18]. A comparable glucose reduction-acid strategy was applied by Lei et al. to spent NCM batteries, where lithium, nickel, cobalt, and manganese were all recovered at rates above 99% [19]. In both cases, the underlying mechanism is the same: glucose reduces high-valence metal ions to lower-valence, more soluble states, accelerating their dissolution, while the sugar itself is gradually oxidized into simpler organic compounds.

Ultrasonic irradiation offers a complementary, environmentally friendly means of intensifying this leaching chemistry. Its benefit stems chiefly from acoustic cavitation: the rapid growth and collapse of microbubbles generates highly reactive hydroxyl radicals [20] alongside localized turbulence, shear forces, shock waves, and microjetting—effects that disrupt the solid–liquid boundary layer and accelerate mass transfer during metal extraction. This advantage has been reported consistently across different leaching systems. In a citric acid H_2_O_2_ system, Cui et al. achieved 96% cobalt recovery and near-complete lithium recovery under ultrasonic assistance [21]. Togonon et al. found ultrasonic stirring particularly beneficial for cobalt recovery, with lithium and cobalt leaching rates of 92.53% and 81.50% after 2 h rising to 99.80% and 96.46% after 5 h [22]. Directly comparing ultrasonic-assisted and conventional leaching under matched conditions, Jiang et al. reported optimal cobalt and lithium recovery of 94.63% and 98.62%, respectively, confirming a clear improvement over conventional stirring [23]. A similar pattern emerged in the work of Wang et al., where mechanical stirring alone in an H_2_SO_4_ system leached lithium efficiently (close to 80%) but left other metals below 50% recovery; switching to ultrasonic stirring raised lithium recovery to 93% [24]. Collectively, these findings indicate that ultrasonic assistance reliably improves leaching performance relative to conventional stirring, though the extent of improvement depends on the specific cathode material and leaching chemistry involved.

Although ultrasonic-assisted leaching has been applied to the recycling of spent LIBs with cathode materials such as LiCoO_2_ and NCM, its application to spent LiFePO_4_ (LFP) batteries remains insufficiently explored. Unlike cobalt-rich cathode materials, LFP presents unique challenges due to the strong covalent P–O bonds within its olivine crystal structure, which significantly hinder the release of Li^+^ and Fe^2+^ during acid leaching. Moreover, most existing studies have not provided a systematic comparison of ultrasonic effects under varying process parameters, nor a comprehensive kinetic analysis specific to LFP systems. To address these gaps, the present study employs H_2_SO_4_ as the leaching agent and glucose (C_6_H_12_O_6_) as a green reducing agent under ultrasonic-assisted conditions to recover Li and Fe from spent LFP cathode materials. The influences of H_2_SO_4_ concentration, C_6_H_12_O_6_ concentration, liquid–solid ratio, leaching temperature, leaching time, and ultrasonic power on the leaching rates were systematically investigated. Furthermore, a shrinking-core kinetic model was applied to elucidate the diffusion-controlled reaction mechanism and determine the activation energies, providing a theoretical basis for process optimization. This work offers a scientifically rigorous and environmentally benign pathway for spent LFP battery recycling and contributes to a deeper mechanistic understanding of ultrasonic-assisted hydrometallurgical processes.

## 2. Experiments

### 2.1. Reagents, Instruments, and Methods of Analysis

Chemical reagents used were all analytically pure and all solutions were prepared using deionized water. Leaching experiments were conducted by taking H_2_SO_4_ solution as the leaching agent and C_6_H_12_O_6_ as the reducing agent.

An ultrasonic cleaner (KQ-100DB, 40–100 W, 40 kHz, Kunshan Ultrasonic Instruments Co., Ltd., Kunshan, China) was used for ultrasonic stirring. An inductively coupled plasma-optical emission spectrometer (ICP-OES) was used to measure the concentration of metallic ions.

### 2.2. Sample Preparation

Spent LFP batteries were obtained from a company in Shenzhen City, China. To eliminate the risk of short-circuiting and fire during subsequent manual dismantling, the batteries were first immersed in 5 wt% NaCl solution for 24 h to allow complete discharge [25]. The discharged batteries were then manually dismantled to take whole cathode and anode electrodes. The cathode electrodes were cut into pieces measuring 10 mm × 10 mm, followed by vacuum pyrolysis at 450 °C for 2 h to remove the organic binder, thus obtaining cathode materials of spent LFP batteries. The cathode powder was subjected to thermogravimetric analysis (TGA) prior to pyrolysis to determine the appropriate binder removal temperature. The TGA curve of the cathode material showed that the sample mass remained essentially unchanged below 300 °C, beyond which a significant weight-loss event was observed, indicating that effective decomposition of the PVDF binder requires temperatures above 300 °C. PVDF is a semicrystalline fluoropolymer that undergoes thermal decomposition via dehydrofluorination and chain scission predominantly in the range of 350–500 °C under inert or vacuum conditions, releasing HF and other gaseous products [26]. A pyrolysis temperature of 450 °C was therefore selected to ensure complete binder removal and reliable detachment of the cathode active material from the aluminum foil current collector, while minimizing energy consumption. The vacuum atmosphere further facilitates the escape of gaseous decomposition products and enhances the overall pretreatment efficiency [27]. These cathode materials were ground and then used as the experimental leaching material. Specimens weighing 25 mg of cathode materials from spent LFP batteries were weighed and dissolved in 10 mL chloroazotic acid to be diluted and reconstructed to 1000 mL. An ICAP-7000 ICP-OES was employed to measure the contents of metals in the cathode materials of spent LFP batteries.

### 2.3. Leaching Experiment

Conventional leaching experiments were conducted in a water bath equipped with a heating device and a constant-temperature magnetic stirrer. In ultrasonic-assisted leaching experiments, an ultrasonic cleaner (KQ-100DB, 40 kHz, 40–100 W) was used. The leaching solution volume was fixed at 45 mL in a 100 mL beaker (inner diameter: 50 mm), which was placed at the center of the ultrasonic tank (internal dimensions: 240 mm × 135 mm × 100 mm). The acoustic energy density ranged from 0.89 W/mL (40 W) to 2.22 W/mL (100 W). The temperature of the leaching solution was monitored in real time using a calibrated thermometer and maintained at the target value via the built-in heating controller of the ultrasonic cleaner throughout each experiment. At first, certain amounts of cathode materials of LFP batteries, C_6_H_12_O_6_, and H_2_SO_4_ were mixed in a 100 mL beaker (the volume fraction of C_6_H_12_O_6_ and H_2_SO_4_ was 1:9), and then the mixture was placed on the magnetic stirrer for stirring and leaching. After a certain period of leaching under different conditions, the solid and liquid were separated through vacuum filtration and samples were collected. The concentrations of valuable metals in the filter liquor were measured using the ICP-OES and the leaching rate (*η*) of valuable metals can be calculated using Equation (1):

(1) $$\eta =\frac{c\times V}{m}\times 100\%$$

where *c* is the concentration of metals in the leaching solution (mg/L); *V* denotes the volume of the leaching solution (L); and *m* is the mass of each element in the cathode materials.

## 3. Results and Discussion

### 3.1. Conventional Reducing Acid Leaching

The aim of reducing acid leaching of cathode materials of LFP batteries using the C_6_H_12_O_6_-H_2_SO_4_ mixed solution is to leach Fe^2+^ and Li^+^ in spent cathode materials to the greatest extent and inhibit the oxidation of Fe^2+^. Influences of the H_2_SO_4_ concentration, C_6_H_12_O_6_ concentration, L/S ratio, leaching temperature, and leaching time on the leaching rate were investigated in the experiments. The single-factor method was utilized to optimize the leaching conditions.

(1) Influence of the concentration of H_2_SO_4_: this was studied at the C_6_H_12_O_6_ concentration of 2 mol/L and temperature of 70 °C with a leaching time of 90 min and an L/S ratio of 10 mL/g.

(2) Influence of the concentration of C_6_H_12_O_6_: this was investigated at the H_2_SO_4_ concentration of 2 mol/L and temperature of 70 °C with a leaching time of 90 min and an L/S ratio of 10 mL/g.

(3) Influence of the L/S ratio: this was studied at the C_6_H_12_O_6_ concentration of 2 mol/L, H_2_SO_4_ concentration of 2 mol/L, and temperature of 70 °C with a leaching time of 90 min.

(4) Influence of temperature: this was explored at the C_6_H_12_O_6_ concentration of 2 mol/L, H_2_SO_4_ concentration of 2 mol/L, and temperature of 70 °C with a leaching time of 90 min and an L/S ratio of 15 mL/g.

(5) Influence of time: this was studied at the C_6_H_12_O_6_ concentration of 2 mol/L, H_2_SO_4_ concentration of 2 mol/L, and temperature of 70 °C, with an L/S ratio of 15 mL/g.

**Figure 1:**
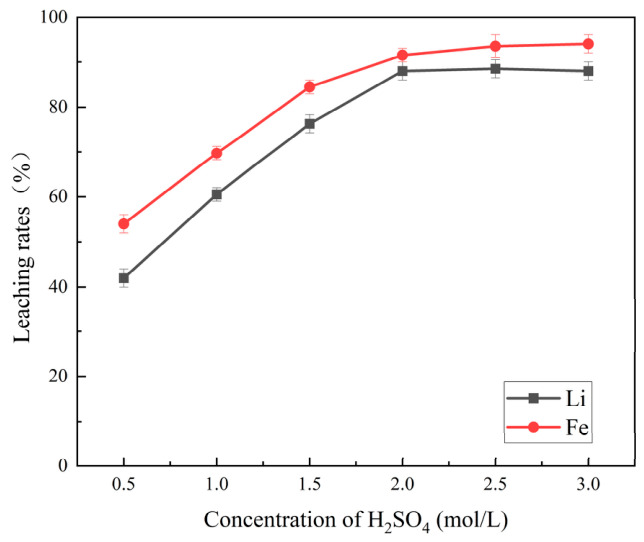
Influence of the concentration of H_2_SO_4_.

**Figure 2:**
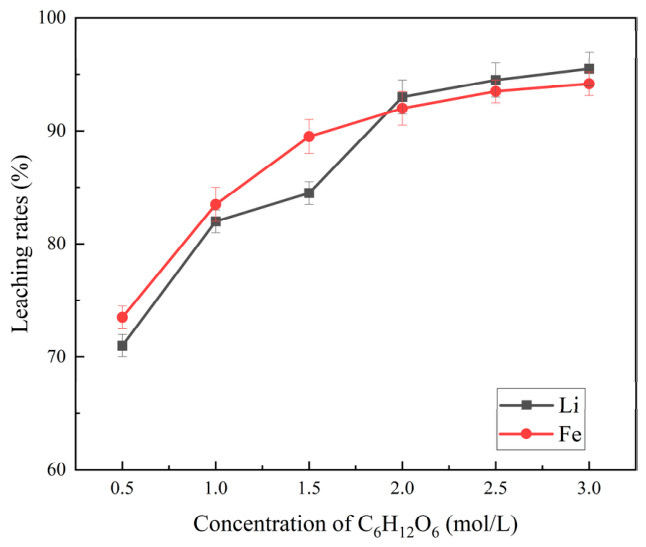
Influence of the concentration of C_6_H_12_O_6_.

**Figure 3:**
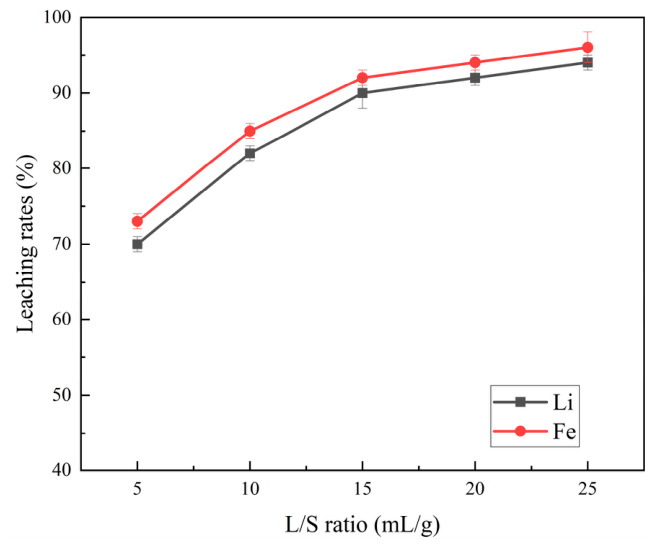
Influence of the L/S ratio.

**Figure 4:**
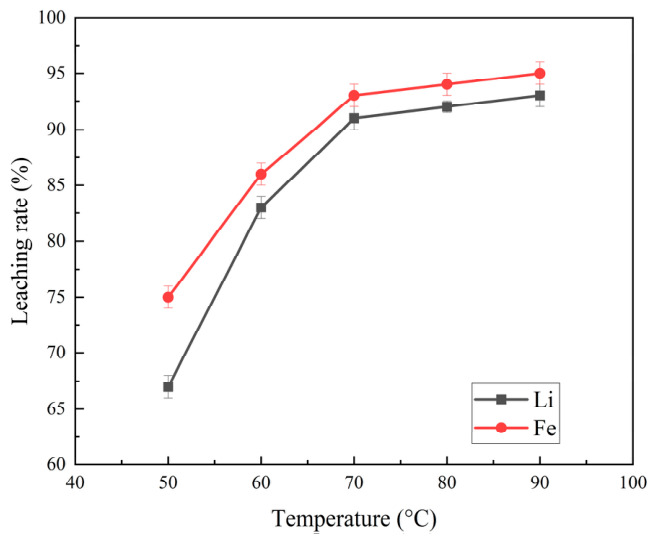
Influence of temperature.

**Figure 5:**
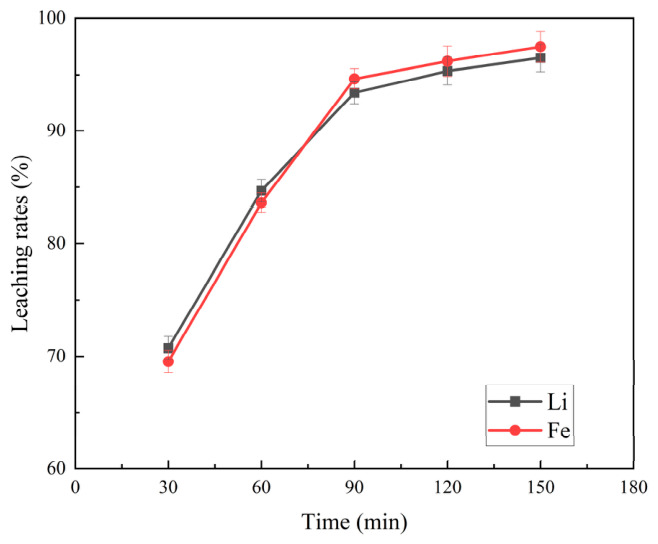
Influence of time.

As shown in Figure 1, Figure 2, Figure 3, Figure 4 and Figure 5, the H_2_SO_4_ concentration, C_6_H_12_O_6_ concentration, L/S ratio, temperature, and time exert different degrees of influences on the leaching process. Figure 1 shows that, as the H_2_SO_4_ concentration is increased, the leaching rates of Li and Fe gradually increase while their growth rates decrease the H_2_SO_4_ concentration exceeds 2 mol/L. When the H_2_SO_4_ concentration is less than 2 mol/L, because the reaction rate is so slow that the reaction does not reach an equilibrium state, increasing the H_2_SO_4_ concentration can provide the H^+^ needed for reaction and promote the forward progress of such reactions, significantly affecting the leaching rate. Once the H_2_SO_4_ concentration is greater than 2 mol/L, the H^+^ concentration is no longer the main factor that controls the reaction. Under such conditions, the influence of further elevating the H_2_SO_4_ concentration on the leaching rate is weakened owing to the principle of chemical equilibrium shift. Therefore, the H_2_SO_4_ concentration of 2 mol/L is selected as an optimized condition. Figure 2 shows that with the growth of the C_6_H_12_O_6_ concentration, the leaching rates of Li and Fe both tend to rise. When the C_6_H_12_O_6_ concentration is low, the reaction fails to reach an equilibrium state at the end of each experiment. Under these conditions, the leaching rates exhibit an incremental trend with increasing C_6_H_12_O_6_ concentration. As the C_6_H_12_O_6_ concentration reaches a certain value, the reaction rate declines and the leaching rate does not increase to any significant extent. It is noteworthy that in LiFePO_4_, iron is present in the +2 oxidation state; therefore, glucose is not stoichiometrically consumed in the primary dissolution reaction but serves to maintain a reducing environment that prevents the re-oxidation of dissolved Fe^2+^. Based on the elemental composition in Table 1 and the experimental conditions (L/S = 10 mL/g, solution volume = 45 mL), the molar ratio of glucose to Fe at 2 mol/L is approximately 3.4:1, confirming that glucose is present in significant excess relative to the iron content. By comprehensively considering the amount of C_6_H_12_O_6_ used and the growth of leaching rates of Li and Fe, the C_6_H_12_O_6_ concentration of 2 mol/L is selected as an optimized condition.

As shown in Figure 3, the L/S ratio increases, the leaching rates of Li and Fe both increase substantially. The increment of the L/S ratio and the reduction in concentration of leaching products accelerate the mass transfer rate in the leaching process and improve the metal leaching rate. Although a higher L/S ratio is more favorable for the leaching reaction, it also elevates the cost. Considering the actual economic benefit, the L/S ratio of 15 mL/g is chosen as an optimized condition.

Figure 4 shows that, as the leaching temperature is increased, the leaching rates of Li and Fe grow rapidly at first. This is because more inactivated molecules are activated with increasing temperature, which increases the reaction rate. The leaching rates are highest at 90 °C, while their growth rates decrease significantly after rising to 70 °C. Therefore, the leaching temperature of 70 °C is selected as an optimized condition.

As shown in Figure 5, the leaching rates of Li and Fe both increase significantly with prolonged leaching time. When the leaching time is 60 min, the leaching rates of Li and Fe are separately 85.69% and 84.47%, while their growth rates decrease when the leaching time exceeds 90 min. The highest leaching rates are found after leaching for 150 min, when the leaching rates of Li and Fe are 96.78% and 97.41%, respectively. Considering that the longer the leaching time, the higher the energy consumption, and that the leaching rates do not grow substantially, the leaching time of 90 min is selected as an optimized condition.

### 3.2. Ultrasonic-Assisted Leaching

To systematically evaluate the enhancement effect of ultrasonic treatment, ultrasonic-assisted leaching experiments were performed under the optimal conditions established in Section 3.1 (H_2_SO_4_ concentration of 2 mol/L, C_6_H_12_O_6_ concentration of 2 mol/L, temperature of 70 °C, and L/S ratio of 15 mL/g), and the resulting leaching rates were directly compared with those of conventional leaching at each corresponding parameter level.

#### 3.2.1. Influence of Time on Ultrasonic-Assisted Leaching

**Figure 6:**
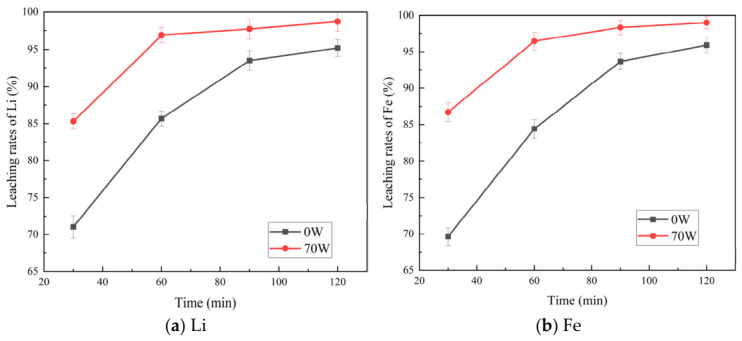
Influence of time on ultrasonic-assisted leaching. (**a**) Li; (**b**) Fe. (At the H_2_SO_4_ concentration of 2 mol/L, C_6_H_12_O_6_ concentration of 2 mol/L, temperature of 70 °C, and L/S ratio of 15 mL/g).

The influence of time on ultrasonic-assisted leaching is illustrated in Figure 6. Under the same condition, the leaching rate of Li after 30 min of ultrasonic treatment is found to increase by 14.6% (from 70.71% to 85.31%) and that of Fe increases by 17.35% (from 69.53% to 86.88%) in comparison with those under conventional leaching. Additionally, the leaching rates of Li and Fe after ultrasonic treatment for 60 min are still higher than those under conventional leaching. This finding implies that ultrasonic treatment can improve leaching rates and the improvement is more significant in the initial leaching stage. The improvement of leaching rates by ultrasonic-assisted leaching is attributed to the ultrasonic cavitation effect [28,29]. During ultrasonic-assisted leaching, ultrasonic waves induce the formation and collapse of bubbles in the liquid and cause violent liquid motion. Such liquid motion may break the liquid–solid boundary layer and continuously expose solid surfaces to the solvent, thus accelerating mass transfer and diffusion. Therefore, ultrasonic-assisted leaching exerts significant influences on the leaching process and the interfacial reaction process.

Figure 6 shows that with the prolonging ultrasonic treatment time (>60 min), the increase amplitudes of leaching rates of Li and Fe are small. After ultrasonic-assisted leaching for 120 min, the leaching rates of Li and Fe are separately 98.9% and 99.13%, which are 3.7% and 3.24% higher than those after conventional leaching for 120 min (with leaching rates of Li and Fe of 95.2% and 95.89%), respectively. The ultrasonic cavitation effect induces particle vibration and collision in samples and enlarges the surface area of solid particles. A larger surface area provides more contact points, enables the solvent to enter the solid more readily and dissolve the solute, which is impossible under conventional mechanical stirring.

Once the ultrasonic-assisted leaching time exceeds 60 min, the leaching rates of Li and Fe only increase slightly, so the leaching time of 60 min is selected as an optimized condition of ultrasonic-assisted leaching.

#### 3.2.2. Influence of Temperature on Ultrasonic-Assisted Leaching

**Figure 7:**
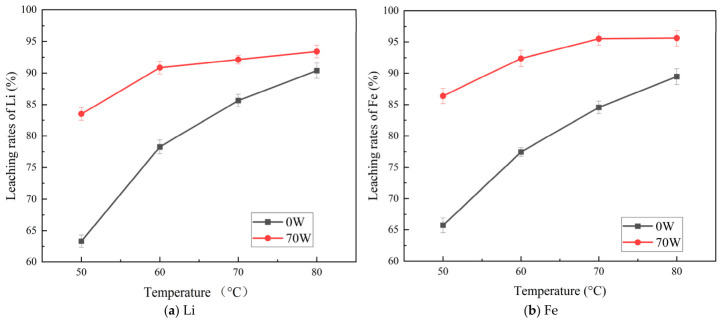
Influence of temperature on ultrasonic-assisted leaching. (**a**) Li; (**b**) Fe. (At the H_2_SO_4_ concentration of 2 mol/L, C_6_H_12_O_6_ concentration of 2 mol/L, and L/S ratio of 15 mL/g).

The influence of temperature on the ultrasonic-assisted leaching effect is displayed in Figure 7: the temperature exerts a significant influence on ultrasonic-assisted leaching. As the temperature is increased, the leaching rates of Li and Fe both gradually increase. At a temperature exceeding 70 °C, the growth rates of leaching rates of Li and Fe decrease. Under the same leaching conditions, the ultrasonic-assisted leaching effect is found to be better than the conventional leaching effect. At a temperature below 70 °C, the leaching rates under ultrasonic-assisted leaching are much higher than those under conventional leaching. At 70 °C, the leaching rates of Li and Fe under conventional leaching are 85.69% and 84.47%, while those under ultrasonic-assisted leaching are 92.36% and 95.39%, respectively. The leaching rates of Li^+^ and Fe^2+^ under ultrasonic-assisted leaching improve by 6.67% and 10.89% compared with those under conventional leaching. At 80 °C, the leaching rates of Li and Fe under conventional leaching are 90.27% and 89.32%, while those under ultrasonic-assisted leaching separately reach 93.02% and 94.92%. The leaching rates of Li and Fe separately improve by 2.75% and 5.6% compared with those under conventional leaching. The results are explained as follows: at a low temperature, the solubility of the gas in liquid decreases with increasing liquid temperature, which allows release of more gas to the liquid, thus increasing the probability of formation of bubbles and enhancing the effects of ultrasonic cavitation; however, once the temperature is too high, the gas content reduces substantially in the liquid, so it is more difficult for ultrasonic waves to form bubbles in the liquid, which weakens the effects of ultrasonic cavitation.

When the temperature exceeds 70 °C, the leaching rates of Li and Fe do not increase to any significant extent either under conventional or ultrasonic-assisted leaching. Therefore, the leaching temperature of 70 °C is selected as an optimized condition for ultrasonic-assisted leaching.

#### 3.2.3. Influence of Ultrasonic Power

**Figure 8:**
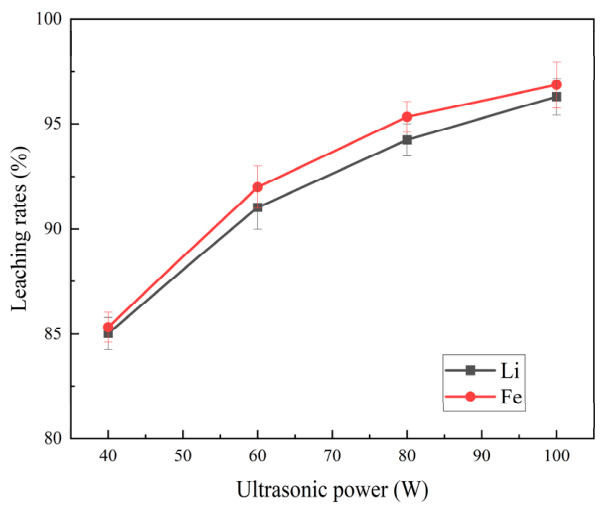
Influence of ultrasonic power on the leaching rates of Li and Fe. (At the H_2_SO_4_ concentration of 2 mol/L, C_6_H_12_O_6_ concentration of 2 mol/L, temperature of 70 °C, and L/S ratio of 15 mL/g).

The influence of ultrasonic power on leaching rates is displayed in Figure 8 when the concentration of H_2_SO_4_ is 2 mol/L, the concentration of C_6_H_12_O_6_ is 2 mol/L, the L/S ratio is 15 mL/g, leaching temperature is 70 °C, and leaching time is 60 min. The figure shows that, when increasing the ultrasonic power, the leaching rates of Li and Fe both increase. Under an applied ultrasonic power of 40 W, the leaching rates of Li and Fe are separately 84.6% and 85.11%. Under an applied ultrasonic power of 100 W, the leaching rates of Li and Fe are 96.53% and 96.8%, which are separately increased by 11.93% and 11.69%. This is mainly because the high-power ultrasonic waves exert a stronger ultrasonic cavitation effect than low-power ones. The number of cavitation bubbles generated by the ultrasonic cavitation effect increases with the increment of ultrasonic power [30]. Ultrasonic cavitation occurs near particle surfaces and cavitation bubble collapse near particle surfaces effectively decreases the thickness of the diffusion layer and enlarges the surface area of particles. With the increase in ultrasonic power, the cavitation effect is enhanced and leaching rates are improved.

### 3.3. Kinetic Study

The leaching process of Li and Fe from cathode materials of spent LFP batteries is a typical liquid–solid reaction, the rate of which is controlled by the chemical reaction or diffusion. As the reaction continues, the powder core of cathode materials shrinks until it almost disappears, which conforms to the shrinking-core model of reactions. The equations for shrinking-core models of chemical reactions and diffusion-controlled reactions are

(2) $$1-{\left(1-X\right)}^{\frac{1}{3}}=k_{c}t$$

(3) $$1-\frac{2X}{3}-{\left(1-X\right)}^{\frac{2}{3}}=k_{d}t$$

where X is the leaching rate (%); $k_{c}$ denotes the rate constant of chemical reactions (min^−1^); $k_{d}$ is the rate constant of diffusion reactions (min^−1^); and *t* is the leaching time (min).

**Figure 9:**
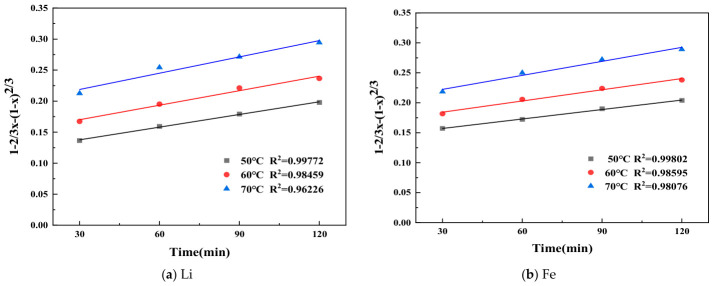
Linear fitting of 1 − 2/3*x* − (1 − *x*)^2/3^ − *t* relationship. (**a**) Li; (**b**) Fe.

Experimental data pertaining to ultrasonic-assisted reducing leaching at different temperatures after different leaching times are fitted with the diffusion-controlled model in Equation (3), and the results are shown in Figure 9.

The correlation coefficients of these lines are approximated to 1, indicating that reaction kinetics are mainly controlled by diffusion of the product layer. The slopes of various lines in the figure are the corresponding reaction rate constants $k_{d}$. The Arrhenius equation is used to calculate the activation energy, as expressed below:

(4) $$\ln k=\ln A-E_{a}/RT$$

where k is the rate constant (min^−1^); *A* represents the frequency factor (min^−1^); *E*_a_ is the activation energy of reactions (J·mol^−1^); and *R* refers to the molar gas constant, which is 8.3145 J·K^−1^·mol^−1^.

The rate-controlling mechanism can be identified by the calculated apparent activation energy $E_{a}$: when $E_{a}<20\;\mathrm{kJ}/\mathrm{mol}$, the process is diffusion-controlled; when $E_{a}>40\;\mathrm{kJ}/\mathrm{mol}$, the process is governed by interfacial chemical reaction [31].

**Figure 10:**
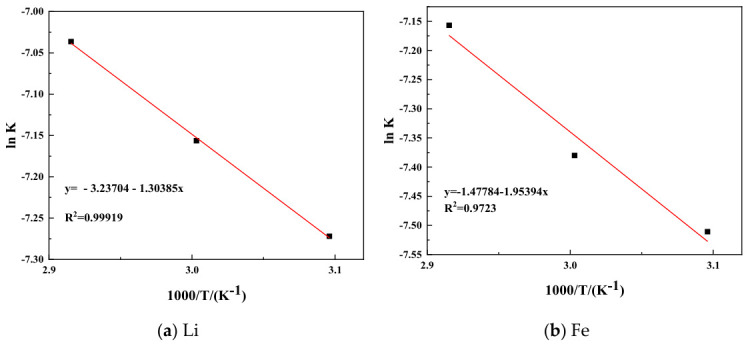
lnk-1000/*T* relationship. (**a**) Li; (**b**) Fe.

Using Equation (4), the lnk-1000/*T* relationship is plotted separately taking the reciprocal of different temperatures and the logarithm of reaction rate constants as abscissa and ordinate, respectively (Figure 10).

Data in Figure 10 are linearly fitted. Letting 1000/T be x and ln k be y, the resulting linear relations of Li^+^ and Fe^2+^ are separately y = −3.23704 − 1.30385x and y = −1.47784 − 1.95394x. The slope of the linear equation reflects the apparent activation energy. The activation energies of Li^+^ and Fe^2+^ are calculated to be 10.84 kJ/mol and 16.24 kJ/mol, respectively, both below 20 kJ/mol, indicating that the leaching process is controlled by diffusion. Lou et al. leached Li from spent LFP batteries in dilute H_2_SO_4_ and obtained an activation energy of 12.69 kJ/mol, also governed by diffusion through the product layer [32]. Niu et al. studied the reduction leaching of iron phosphate residue in H_2_SO_4_ with a reducing agent and reported an activation energy of 12.71 kJ/mol under diffusion control [16]. By contrast, leaching systems without ultrasonic assistance tend to exhibit higher activation energies, reflecting greater mass transfer resistance. Liu et al. conducted H_2_SO_4_ leaching of LFP without ultrasonic assistance and obtained an activation energy of 22.99 kJ/mol, attributed to mixed surface-diffusion control [17]. The lower activation energies in the present study indicate that ultrasonic cavitation thins the diffusion boundary layer and continuously exposes fresh solid surfaces, reducing the energy barrier for mass transfer [33]. It is acknowledged that the Arrhenius analysis is based on three temperature data points, which represents a limitation of the present kinetic study. Nevertheless, the high R^2^ values obtained for the linear fitting (R^2^ = 0.99919 for Li and R^2^ = 0.9723 for Fe) indicate a statistically acceptable fit, and the calculated activation energies are consistent with independently reported values for analogous leaching systems, supporting the reliability of the diffusion-controlled mechanism identified in this work.

### 3.4. Mechanism of Ultrasonic-Assisted Reducing Leaching

**Figure 11:**
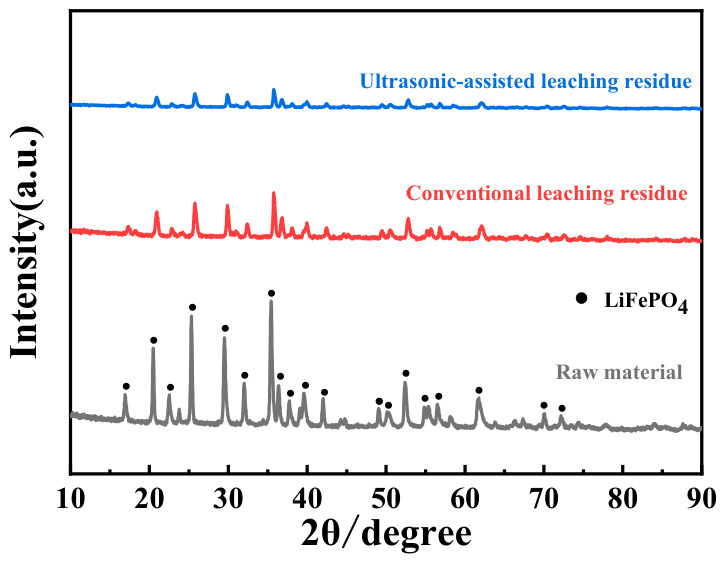
X-ray diffraction patterns of the ultrasonic-assisted leaching residue, conventional leaching residue, and raw material.

Figure 11 shows the X-ray diffraction (XRD) patterns of the ultrasonic-assisted leaching residue, conventional leaching residue, and raw material. The ultrasonic-assisted leaching residue and conventional leaching residue share the same phase compositions with the raw material, all composed of LiFePO_4_, and no other phases are detected. Compared with conventional leaching, the intensity of diffraction peak of LiFePO_4_ under ultrasonic-assisted leaching decreases obviously, which is a result of the incomplete reaction of LiFePO_4_ under conventional leaching.

**Figure 12:**
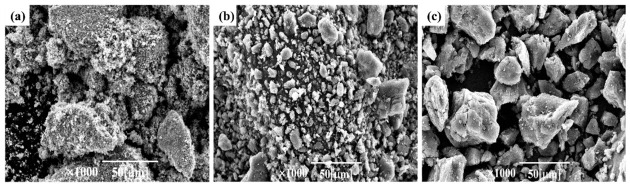
SEM images of the raw material (**a**), ultrasonic-assisted leaching residue (**b**), and conventional leaching residue (**c**).

Figure 12 illustrates the SEM images of the raw material, conventional leaching residue, and ultrasonic-assisted leaching residue. In Figure 12a, the raw material consists of nano-sized primary particles that form agglomerates of 20–30 μm. After leaching, the agglomerate size is significantly reduced. As shown in Figure 12b,c, the agglomerates after ultrasonic-assisted leaching are much smaller than those after conventional leaching, with most agglomerates reduced to below 1 μm, compared to approximately 10 μm under conventional leaching. This indicates that ultrasonic cavitation effectively breaks down the agglomerates and shortens the diffusion path length. According to the shrinking core model, the leaching rate under diffusion control is inversely proportional to the square of the particle radius; thus, the observed reduction in agglomerate size provides physical evidence consistent with the diffusion-controlled mechanism identified in the kinetic analysis [34]. Ultrasonic stirring not only promotes convective motion, enlarges the solid–liquid contact area, and accelerates leaching rate, but also provides huge energy and facilitates dissolution of the raw material. After bubbles generated under the unique ultrasonic cavitation effect burst, the transient local high temperature and high pressure generated damage the solid surface, which is conducive to improving the leaching rate. The results show that the ultrasonic-assisted leaching process of Li and Fe from spent LFP batteries is controlled by diffusion. Ultrasonic stirring facilitates mass transfer and diffusion through the ultrasonic cavitation effect and mechanical effect.

### 3.5. Comparison with Reported Ultrasonic-Assisted Leaching Studies

To further evaluate the performance of the proposed process, a comparison with previously reported ultrasonic-assisted leaching systems for spent lithium-ion batteries was conducted. Jiang et al. reported that ultrasound-assisted leaching using H_2_SO_4_ and H_2_O_2_ achieved 98.21% Co and 99.15% Li recovery from spent LiCoO_2_ cathodes within 60 min, demonstrating the significant enhancement of mass transfer and diffusion by ultrasonic cavitation [23]. Likewise, Esmaeili et al. employed lemon juice combined with H_2_O_2_ under ultrasonic conditions and obtained nearly complete Li recovery together with approximately 96% Co and Ni extraction [35]. More recently, Yan et al. utilized mixed organic acids and biomass-derived reductants in an ultrasonic-assisted system and achieved over 95% recovery of Li, Ni, Co, and Mn under relatively mild conditions [36]. Compared with these studies, the present ultrasonic-assisted H_2_SO_4_–glucose reductive leaching process achieved Li and Fe leaching efficiencies of 96.53% and 96.80%, respectively, at 70 °C within 60 min. Furthermore, glucose served as a low-cost and environmentally friendly reducing agent, avoiding the use of additional oxidants or hazardous reducing chemicals. These results indicate that the proposed process exhibits competitive leaching performance while providing advantages in terms of sustainability, reagent safety, and potential industrial applicability.

From an industrial perspective, the additional energy consumption associated with ultrasonic treatment should also be considered when evaluating the practical applicability of the proposed process. In this study, ultrasonic irradiation was performed at a relatively low power of 100 W for 60 min. Although ultrasound introduces additional energy input, the cavitation effect significantly enhances mass transfer and accelerates the dissolution of LiFePO_4_, resulting in high leaching efficiencies within a relatively short reaction time. The improved kinetics may reduce overall processing time and potentially decrease reactor volume requirements in industrial applications. Therefore, the benefits associated with enhanced leaching performance may partially offset the additional energy consumption. Nevertheless, a comprehensive techno-economic assessment, including detailed energy consumption analysis, will be necessary in future studies to fully evaluate the industrial feasibility of the process.

## 4. Conclusions

(1) The leaching time, leaching temperature, the H_2_SO_4_ concentration, the C_6_H_12_O_6_ concentration, L/S ratio, and ultrasonic power all affect the leaching rates of Li and Fe from spent LiFePO_4_ to some extent. Under optimal conditions (leaching time of 60 min, temperature of 70 °C, H_2_SO_4_ concentration of 2 mol/L, C_6_H_12_O_6_ concentration of 2 mol/L, L/S ratio of 15 mL/g, and ultrasonic power of 100 W), the leaching rates of Li and Fe reach 96.53% and 96.8%, respectively. Compared with conventional leaching, the introduction of ultrasonic treatment under the same conditions greatly improves the leaching rates of Li and Fe, and the time required to achieve optimal leaching rates is significantly shortened. These results demonstrate that integrating ultrasonic cavitation with a green glucose reducing agent constitutes an efficient and environmentally benign alternative to conventional hydrometallurgical recycling of spent LFP batteries.
(2) The leaching rates under high-power ultrasonic-assisted leaching are higher than those under low-power ultrasonic-assisted leaching. The phase compositions of the ultrasonic-assisted leaching residue and conventional leaching residue are the same as those of the raw material, all composed of LiFePO_4_, and no other phases are found. This selectivity in dissolution, achieved without inducing unwanted phase transformations, suggests that the proposed approach holds broader applicability for the selective recovery of critical metals from other spent lithium-ion battery cathode chemistries.
(3) Kinetic analysis of ultrasonic-assisted reducing leaching shows that the activation energies of Li and Fe are 10.84 kJ/mol and 16.24 kJ/mol, respectively, confirming that the leaching process is diffusion-controlled. The established kinetic framework provides a mechanistic foundation for the rational design and scale-up of ultrasonic-assisted hydrometallurgical processes. Future research should focus on the selective separation and purification of recovered Li and Fe for direct precursor resynthesis, as well as pilot-scale validation to assess the industrial feasibility of this process and its extension to other spent cathode materials. Furthermore, future studies employing response surface methodology (RSM) or complete factorial design would be worthwhile to capture the interaction effects among leaching parameters and achieve more comprehensive process optimization.

## Figures and Tables

**Table 1:** ICP-OES analysis of cathode materials of spent LFP batteries (%mass fraction).

Composition	Li	Fe	P	Al	Others
Contents	4.36	33.27	18.45	0.067	43.853

## Data Availability

The original contributions presented in this study are included in the article. Further inquiries can be directed to the corresponding author.
